# Development of Polymer–Gel Fibrous Composites for Well Water Shutoff in Fractured–Porous Carbonate Formations

**DOI:** 10.3390/polym17111541

**Published:** 2025-06-01

**Authors:** Aleksey Telin, Ravil Yakubov, Artem Pavlik, Vladimir Dokichev, Rida Gallyamova, Anton Mamykin, Farit Safarov, Vladimir Strizhnev, Sergey Vezhnin, Anatoly Politov, Lyubov Lenchenkova

**Affiliations:** 1 Ufa Scientific and Technical Center, LLC, 99/3, Kirova Street, 450078 Ufa, Russia; mamikinaa@ufntc.ru (A.M.);; 2 Faculty of Mining and Petroleum, Ufa State Petroleum Technological University, 1, Kosmonavtov Street, 450064 Ufa, Russia; 3 Ufa Institute of Chemistry, Ufa Federal Research Center, Russian Academy of Sciences, 71, Oktyabrya Avenue, 450054 Ufa, Russiarida_gallyamova@mail.ru (R.G.); 4 Institute of Solid State Chemistry and Mechanochemistry of Siberian Branch RAS, 18, Kutateladze Street, 630128 Novosibirsk, Russia

**Keywords:** well water shutoff, fractured carbonate reservoirs, polymer–gel fibrous composites, elasticity modulus, residual resistance factor, field well tests

## Abstract

The challenge of water shutoff in carbonate reservoirs is complicated by the presence of fractures, which cannot be effectively blocked using conventional hydrogel screens designed for granular reservoirs. To reliably seal fractures, fibrous and dispersed fillers are added to hydrogels. These fillers must exhibit affinity for the matrix to ensure the composites can effectively isolate water. Given the wide variability in fracture apertures, it is evident that water shutoff composites should incorporate fibers and dispersed fillers of varying geometric sizes. This study presents a range of hydrogel composites reinforced with mono-, bi-, and tri-component fibers, as well as dispersed fillers, designed for water shutoff in fractured carbonate reservoirs with varying fracture apertures. Oscillation test results demonstrated a twofold increase in the elastic modulus (40–45 Pa) for compositions with various fillers compared to the base composition (23 Pa). Filtration studies revealed the effectiveness of the optimized compositions under different fracture apertures. Specifically, even at a fracture aperture of 650 μm, the residual resistance factor (RRF) reached 82.3 and 9.76 at water flow rates of 0.1 cm^3^/min and 0.5 cm^3^/min, respectively. The conducted rheological and filtration tests, along with field trials, confirmed the validity of the selected approach.

## 1. Introduction

Fiber-reinforced polymer composites currently represent one of the most promising and rapidly developing areas in polymer science and technology, with applications spanning virtually all aspects of human activity [1]. The application of composite materials based on polymers reinforced with synthetic and natural fibers has become firmly established in the oil industry over the past 30 years, being used in nearly all stages of oil production processes. Let us note just some of the most significant aspects of fibrous composite applications: cementing materials for well construction and workovers [2,3,4]; fiberglass pipes that match the strength of carbon steel pipes while being resistant to corrosion [5,6]; fiberglass liner systems for isolating leaks in extended-length production casings [7]; inflatable composite sleeves using elastomers, thermosetting resins, and fibers to eliminate casing leaks with minimal diameter reduction [8]; water inflow isolation in wells using fiber-reinforced cement slurries with thermoplastic polymer fibers [9]; blocking compositions for well killing operations [10,11]; acid-diverting compositions for matrix acid treatments [12,13]; completion fluids for well finishing [14]; conformance control compositions for injection profile equalization (the monograph [15] summarizes the experience of using polymer–fiber systems for well injectivity profile conformance in Russia, while the studies [16,17] describe experimental research on the application of fibrous composites under various mining and geological conditions); and fracturing fluids [18,19].

The listed applications of fiber–polymer composites, while not claiming to be exhaustive, demonstrate the broad potential of composite materials in the oil industry. We should also mention the review [20] devoted to the use of natural fiber-based composites in the oil and gas sector.

Let us examine in more detail the application of polymer–gel–fiber composites for well water shutoff. In the early 2000s, studies began emerging on the use of fiber–gel composites to shut off watered-out zones and isolate water in fractured reservoirs with low formation pressure. For example, the paper [21] describes the successful application of polyacrylonitrile fiber (PANF) in a gel-forming sediment composite for zone isolation under well lost circulation conditions.

The use of cellulose nanofibers in hydrogel matrices of various compositions for well water shutoff is driven by their ability to be produced from inexpensive industrial cellulose while also being renewable natural materials. For instance, study [22] incorporated cellulose nanofibrils into silica gel to enhance gel strength through reinforcement. Paper [23] developed a robust polyvinyl alcohol/cellulose nanofiber gel for water control, which strengthens through water diffusion. In the article [24], a gel of 0.5% xanthan and 3% cellulose fiber dispersion in a 5% NaCl solution was proposed for well water shutoff. A self-healing nanocellulose gel grafted with polyacrylic acid and crosslinked with Fe^3+^ demonstrates high elasticity and capability to isolate liquid CO_2_—a significantly more challenging task than water isolation [25]. A straightforward technical solution was proposed in work [26], where the microfiber component was produced through hydrolysis of plant-based cellulose feedstock combined with dolomite powder and potassium dichromate. However, the use of potassium dichromate makes this composite environmentally unsuitable for well applications.

An innovative solution involving pre-formed gel particles combined with fibrous materials for reliable water shutoff in fractures was proposed in study [27]. Experimental work [28] describes the use of a treatment fluid containing 1.5% cellulose fibers (100–200 μm) in a 0.4% xanthan solution for fractured reservoirs, reinforced by subsequent injection of crosslinked polymer gel. The treatment is completed with a permeability-restoring fluid—an enzyme breaker—which maintains matrix permeability while effectively sealing the fractures.

Fiber-reinforced composites for enhanced well water shutoff in fractured–porous reservoirs are also fabricated using thermosetting and epoxy resins, with their curing time being controlled by reservoir temperature and crosslinker additives [29,30].

We have successfully applied composites based on polyacrylamide gel, polypropylene fiber (PPF), and chrysotile microfibers to eliminate severe lost circulation during drilling operations [31]. The approach most similar to ours was developed by Tatneft PJSC for well water shutoff: their technology uses a gel matrix composed of polyacrylamide and chromium acetate, reinforced with fibers such as fiberglass, basalt fiber (BF), and PPF, with fiber content ranging from 0.05% to 0.3%. Additionally, zinc oxide or magnesium oxide is added as a binding filler. The treatment fluid volume per well ranges from 30 to 100 m^3^, and the technology has been implemented since 2014 [32].

Prior to conducting water shutoff operations, it is crucial to have data on fracture density and aperture, as it is known that fracture apertures in such reservoirs can vary widely: from 5 to 500 μm [33]. For instance, at the Logovskoye field (Perm Krai), fracture apertures determined by three independent methods range from 5 to 12 μm [34]. On the Yurubcheno-Tokhomsk oil and gas condensate field in Eastern Siberia, fracture apertures in the Riphean reservoir range from 100 to 400 μm, averaging approximately 300 μm [35]. Given this variability, composite materials must incorporate fibers with corresponding thicknesses. The article [36] notes that wells intersecting concentric fractures respond more significantly to water shutoff treatments, highlighting the need to account for fracture orientation and structure when planning well interventions. Furthermore, fracture permeability—depending on orientation, density, and aperture—can reach values as high as several tens of Darcy [37].

All these characteristics of carbonate reservoirs require service companies specializing in water shutoff to maintain a portfolio of composite materials capable of addressing the challenges posed by complex reservoir development. The composites must effectively reduce fracture permeability while possessing high structural–mechanical properties to withstand hydrodynamic water pressure during well production.

It is well established that strengthening fiber-reinforced composites requires hydrogen bonding or van der Waals forces between the polymer matrix and fibrous filler. In this case, the interfacial adhesion energy must exceed the matrix’s cohesive energy. To achieve this, various methods for modifying the surface of carbon fibers (CFs) are employed. Composites incorporating modified CF often demonstrate enhanced strength [38].

In polyacrylamide hydrogel matrices, the addition of 9% nanosilica particles increases hydrogel strength by 5000% through the formation of multiple hydrogen bonds between the silica and the polymer’s functional groups [39,40]. Hydrophobic nanoparticles interact with the main polymer chain via van der Waals forces, further enhancing hydrogel strength [41]. This demonstrates that incorporating nano- and microdispersions into fibrous composites is an effective method for improving the structural–mechanical properties of sealing materials.

To summarize the literature review, another critical consideration is that effective water isolation in fractures requires a gel based on partially hydrolyzed polyacrylamide crosslinked with organic agents. This is because covalently crosslinked gels exhibit superior structural–mechanical properties and thermal stability compared to gels crosslinked with Cr^3+^ or Al^3+^ ions [42].

The aim and scientific novelty of this work consist in developing a series of insulating composites (with fibrous and dispersed fillers) suitable for virtually all possible fracture apertures in fractured–porous oil-saturated reservoirs.

## 2. Materials and Methods

### 2.1. Gel and Additives

For the preparation of the base matrix (BM), consisting of partially hydrolyzed polyacrylamide (PAM) (1.7%) and a complex crosslinking agent composed of paraform (0.15%) and resorcinol (0.05%), the following materials were used: PAM-grade EOR-1141 mass fraction of the main component (MFMC) 98% (TU 20.59.59-001-28618428-2018, LLC “KhimInTek”, Perm, Russia) with molecular weight of 3 × 10^6^ g/mol and hydrolysis degree of 30%; paraformaldehyde MFMC 95% (TU 6-09-141-03-89 with amendments 1, 2); technical-grade resorcinol; 1st-class MFMC 98.5% (OKP 2472110130, JSC “Uralkhimplast”, Nizhniy Tagil, Russia).

The rationale for optimal component concentrations in the base hydrogel was addressed in our earlier studies [43,44,45,46]. The polyacrylamide/crosslinker ratios were optimized according to specific application targets: enhanced oil recovery (gel–polymer flooding), injection profile conformance, or water/gas shutoff. These formulations (base matrix) have been successfully implemented by our team over the past decade.

The base gel used in this study is tailored for moderate temperature conditions (+16 to +40 °C). For other temperature ranges, we have developed modified compositions using the same core components with gelation retarders or accelerators. While minor adjustments may be required for extreme reservoir temperatures (high or low), the fundamental polyacrylamide/crosslinker ratio in fiber-reinforced composites will remain largely unchanged.

As fibrous fillers, products from Russian manufacturers were used, namely PAN fiber (GOST R 51626-2000, LLC “Alabuga-Fiber”, Elabuga, Russia); PP fiber (Atren-Fibre, TU 2458-029-63121839-2011, LLC “MKO”, Kazan, Russia); chopped basalt fiber (LLC “Kamenny Vek”, Dubna, Russia); carbon-fiber-grade UMT (GOST R 57407-2017, LLC “Alabuga-Fiber”, Elabuga, Russia), carbon fiber oxidized with nitric acid; carbon fiber with SiO_2_ deposited by the sol–gel method; carbon fiber with SiO_2_ deposited by the electrochemical method.

The following dispersed fillers were employed: zinc oxide MFMC 99.7% (GOST 202-84, median particle size—689 nm, LLC “STIL”, Izhevsk. Russia) and mechanically activated shungite (median particle size—12.77 µm, Karelia, Russia).

As microfibers, chrysotile asbestos was used (GOST 12871-2013, PJSC “Uralasbest”, Asbest, Russia), representing hollow tubes with outer diameter 30–40 nm, inner diameter 5–11 nm, and length 14–30 μm [47].

All fibrous materials had fiber lengths of 3–6 mm, determined by the design features of equipment used for composite injection into formations, particularly bottomhole assembly and dual-packer tool configurations. Fiber thickness specifications were as follows: PANF—2 μm; PPF—4 μm; BF—5 μm; CF—2 μm; CF with sol–gel-deposited SiO_2_—3 μm; CF with electrochemically deposited SiO_2_—4 μm; oxidized CF—4 μm.

The surface of carbon fibers was examined using the Hitachi Regulus 8220 (Hitachi High-Tech Corporation, Tokyo, Japan) and Tescan Vega 3LMN (Tescan, Brno - Kohoutovice, Czech Republic) scanning electron microscopes (SEMs) in secondary electron mode. The IR spectra of CFs were obtained by attenuated total reflection (ATR) method with diamond elements at a scanning frequency of 4 cm^−1^ using an FT-805 Fourier spectrometer (Simex, Novosibirsk, Russia).

Numerous methods exist for modifying carbon fibers [48,49]. Nitric acid oxidation and silicon dioxide coating are among the simplest and most convenient approaches for producing carbon fibers with either hydrophilic or hydrophobic surfaces. These methods face no chemical or technological limitations. The required reagents are commercially available, and no carbon fiber degradation occurs.

Nitric acid oxidation generates hydroxyl, carbonyl, and carboxyl groups, which promote hydrogen bonding between the polymer matrix and fibrous filler in composite materials while also enhancing adhesion to SiO_2_ coatings.

We hypothesize that applying SiO_2_ coatings to carbon fiber surfaces—similar to incorporating nanosilica particles into polyacrylamide hydrogel matrices—may increase hydrogel strength through multiple hydrogen bonds between siloxane groups and polyacrylamide functional groups.

Surface modification of CF with 65% nitric acid (high purity) [50,51] was carried out according to the following procedure: 0.5 g of CF was placed in a round-bottom flask and 80 mL of 65% nitric acid was added. The mixture was maintained in a water bath at 85 °C for 2 h. The fibers were then extracted from the acid, washed with distilled water, and dried in a drying chamber at 85 °C for 20 min.

SiO_2_ coating was applied using two methods: dip-coating method and electrochemical deposition method.

The dipping method involved the following procedure. The nitric acid-treated carbon fibers with a length of 6–8 cm were immersed in a sol–gel solution (composition specified in reference [52]) and held for 15 s. The fibers were then removed from the solution and air-dried for 30 min in a vertical position. Subsequently, the coated fibers underwent thermal treatment at 500 °C, with a heating rate of 2.5 °C/min to reach the target temperature. The fibers were maintained at this temperature for 30 min.

The electrochemical deposition method involved the following procedure. Carbon fibers 6 cm in length were wrapped on one end with aluminum foil and immersed in a sol–electrolyte solution [53]. A graphite electrode served as the anode, while the CF functioned as the cathode. The anode and cathode were connected to a laboratory power supply (UTP1306S) operating at a current density of 7 mA/cm^2^ for a deposition time of 3 min. The coating formed via an electrochemical reaction, with particles depositing onto the CF surface.

**Figure 1:**
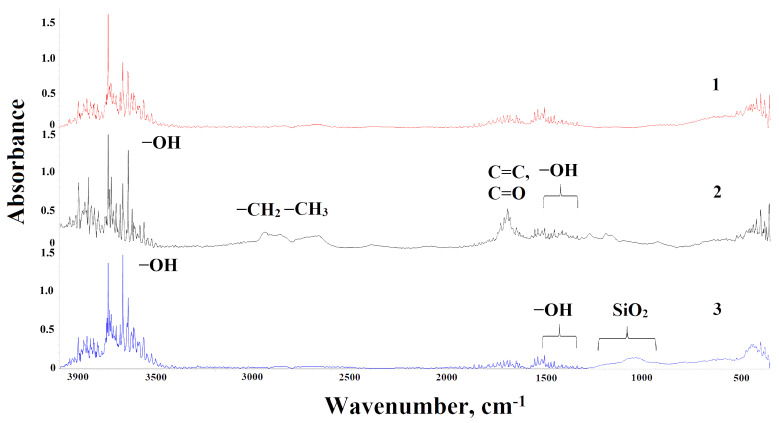
IR spectra of CFs: 1—CF; 2—CF treated with nitric acid; 3—CF with SiO_2_ coating (obtained by dip coating method).

The presence of functional groups on the surface of the studied CFs was determined by ATR-FTIR spectroscopy (Figure 1). For all CF samples, absorption bands were observed in the 3970–3400 cm^−1^ region, characteristic of the stretching vibrations of hydroxyl groups, including those belonging to carboxyl groups. The presence of OH-groups on the fiber surface is also evidenced by bands in the 1500–1350 cm^−1^ range, corresponding to deformation vibrations of hydroxyl groups.

For CF treated with 65% nitric acid, intense broad bands of methyl groups (-CH_3_) and methylene groups (-CH_2−_) were observed in the 2880–2860 cm^−1^ and 2935–2915 cm^−1^ ranges, respectively, which disappear upon application of SiO_2_ coating. The oxidized sample showed increased intensity of bands related to C=C and C=O bonds (1750–1640 cm^−1^). A broad spectrum of low-intensity peaks in the 1250–975 cm^−1^ region indicates the presence of various silica groups on the surface of CFs with SiO_2_ coating.

**Figure 2:**
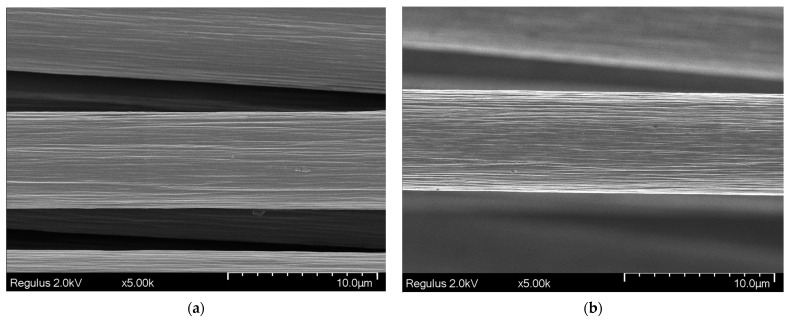
SEM images of carbon fiber: (**a**) after sizing removal; (**b**) after HNO_3_ treatment.

Figure 2 shows the surface of CF in its initial state and after treatment with HNO_3_. Following nitric acid treatment, the fiber surface becomes more textured, with the characteristic longitudinal striations running along the fiber axis appearing more pronounced.

**Figure 3:**
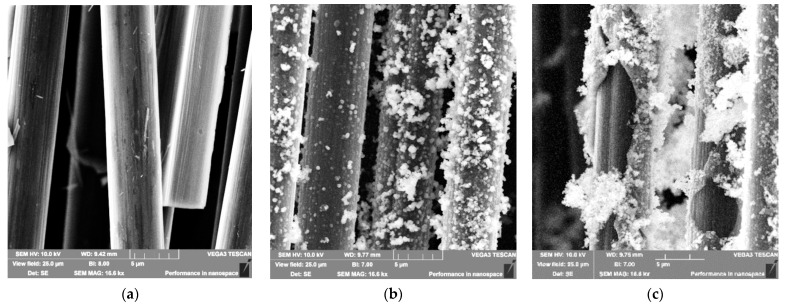
SEM images of carbon fiber with SiO_2_ coating: (**a**)—dip-coating method; (**b**) and (**c**)—electrochemical deposition method.

Figure 3 shows SEM images of fibers with deposited SiO_2_ coating obtained by scanning electron microscopy. The coatings formed uniformly on the fibers, although some areas lacked the SiO_2_ oxide layer. The dip-coating method produced an SiO_2_ coating layer with thickness below 30 nm. The coating thickness on carbon fibers obtained by electrochemical deposition measured 447 ± 168 nm. The coating continuity achieved by dip-coating and electrochemical deposition methods was 35.0 ± 1.0% and 73.0 ± 1.7%, respectively.

The coating thickness of the samples was measured using the obtained SEM images. Between 20 and 30 measurements were performed for each sample. SEM images showing coating fractures or cracks enabling thickness measurements were selected. The SEM analysis and thickness measurements were conducted according to the methodology described in reference [53].

As a dispersed additive in gel–fiber composites, we used shungite mineral obtained from the Zazhoginskoye deposit (Karelia, Russia) with the following composition (wt.%): C (30), SiO_2_ (57), TiO_2_ (0.2), Al_2_O_3_ (4.0), FeO (0.6), Fe_2_O_3_ (1.49), MgO (1.2), MnO (0.15), K_2_O (1.5), and S (1.2). Based on the C/Si ratio, our mineral belongs to type II shungite (shungite II), with estimated deposits of 35 billion tons in Karelia (Russia) and annual industrial production of 200,000 tons [54]. Shungite rocks consist of globular shungite carbon and highly dispersed silicate phases. The globular carbon comprises nanostructures that form larger crystallites within shungite’s amorphous matrix, typically 1 to several microns in size [55]. This unique structure gives the natural mineral a relatively low specific surface area (2–15 m^2^/g) [56]. However, the surface area can be significantly enhanced through thermal and mechanochemical modification. As demonstrated in [57], mechanochemical treatment increases shungite’s specific surface area 35 times (up to 70.6 m^2^/g) while reducing the average particle size to 200–300 nm. These shungite nanoparticles provide substantially increased contact surface with the dispersion medium.

**Figure 4:**
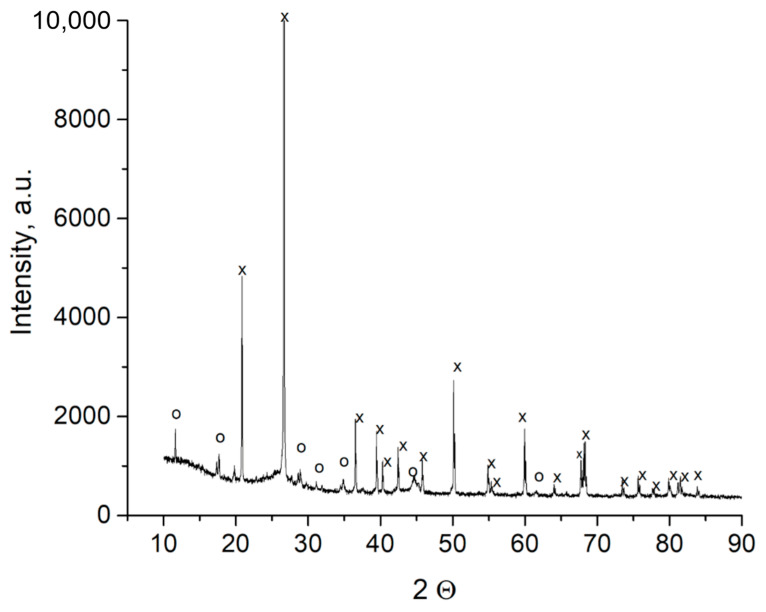
X-ray diffraction pattern of ground shungite (x—diffraction reflection of quartz, o—diffraction reflection of alumosilicates).

To enable shungite dispersion in composites, mechanical activation was performed. However, this process presented specific challenges due to the carbon present in shungite acting as an excellent lubricant, significantly reducing the friction coefficients of the processed powder. The low friction between the drum walls, balls, and ground powder alters the operation mode of the mechanochemical reactor, preventing effective mechanical activation in the impact regime (the most efficient mode for grinding and activation) in planetary mills. Consequently, shungite was mechanically treated in an APF-3 mill (APF-3: Planetary Friction Activator, developed at the Institute of Solid State Chemistry and Mechanochemistry SB RAS, Novosibirsk, Russia) in friction mode for 30 min. X-ray diffraction analysis of the ground shungite was conducted using a D8 Advance diffractometer (Bruker AXS GmbH, Karlsruhe, Germany) with λ = 1.54 Å (Cu Kα radiation). Most observed peaks were reliably identified as quartz reflections (Powder Diffraction File Database: PDF No. 040-05-4718) [58]. Weak signals marked by circles on the diffractogram could not be definitively identified, and may correspond to feldspars and clays present in shungite (Figure 4).

The dispersed and fibrous fillers were dissolved in fresh water using a magnetic stirrer for 10–15 min, followed by the addition of the polymer and crosslinking agent. The resulting mixture was stirred for 45 min or until complete polymer dissolution. After 48 h, oscillatory and filtration tests were conducted.

### 2.2. Oscillatory Rheology Studies

**Figure 5:**
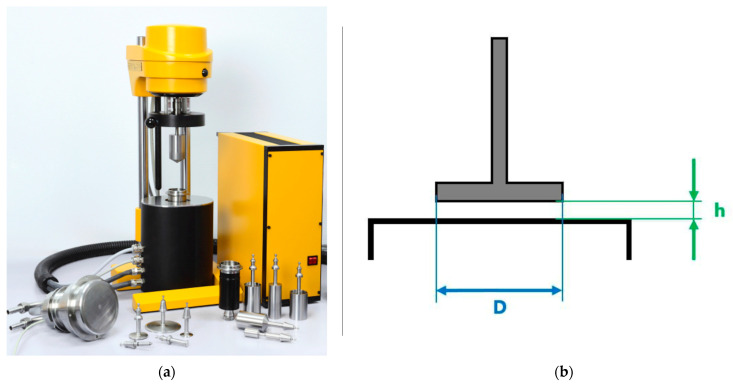
Rotational viscometer Rheotest RN 5.1: (**a**) viscosimeter image; (**b**) plate-plate measuring system (D—plate diameter, h—gap between plates).

Oscillatory measurements were performed using a Rheotest RN5.1 rotational viscometer (Rheotest Medingen GmbH, Ottendorf-Okrilla, Germany) equipped with a “plate-plate” measuring system at 24 °C. The measuring plate diameter (D) was 36 mm, and the gap between plates (h) was set to 1 mm (Figure 5).

Standard measuring geometries for oscillatory studies of hydrogels typically include cone–plate and parallel-plate configurations, as they preserve sample structure during loading. However, the cone–plate geometry has a minimal central gap of only tens of micrometers, making it suitable only for systems with dispersed fillers at least 10 times smaller than this gap size. In our case, the dimensions of dispersed/fibrous fillers substantially exceeded this limit, so the parallel-plate system was selected. A 1 mm gap was used, which represents the standard setting recommended by the instrument manufacturer.

All measurements were performed at 24 °C (laboratory ambient temperature). This temperature was maintained consistently throughout the comparative study of structural–mechanical properties of hydrogels containing various fillers to ensure data comparability.

The required volume of hydrogel was applied to the plate using a dosing syringe, followed by gap adjustment to 1 mm using a micrometer. Excess hydrogel was removed with specialized tweezers to ensure complete filling of the inter-plate space with test fluid.

Tests were performed with a stress sweep (τ) at a fixed oscillation frequency (ν) of 1 Hz. Key measured parameters included the storage (elastic) modulus (G′), loss (viscous) modulus (G″), complex modulus (G*) and crossover point (intersection of G′ and G″ curves), corresponding to the yield stress, as well as the linear viscoelastic region (LVR). Multiple measurements were performed for each sample, with subsequent averaging of results and calculation of standard deviations.

The LVR is the linear viscoelastic domain that plays a crucial role in oscillatory rheological testing of viscoelastic gels. In this region, stress is directly proportional to strain, meaning the material exhibits elastic behavior without structural breakdown. This enables investigation of the gel’s internal structure without causing damage.

The elastic modulus (G′) and viscous modulus (G″) values for the studied gels were obtained specifically within this region. Comparison of LVR ranges allows evaluation of elastic properties across different gels and provides insight into their structural strength and stability.

### 2.3. Low-Pressure Filter Press Testing

Filtration tests were conducted in an FLR-2 filter press (Production Enterprise “OMA”, Ufa, Russia) (156 mL cell) at 0.7 MPa and 20 °C using a slotted filter (300 µm × 30 mm). The 48 hr cured slurry (150 mL) was tested, and filtrate volume was recorded after 30 min.

### 2.4. Particle and Fiber Size Determination

Particle size distribution and average particle size were determined using a SALD-2300 laser diffraction analyzer (Shimadzu, Kyoto, Japan) equipped with a SALD-MS23 flow cell, enabling measurements in the 17 nm to 2500 μm range.

Fiber diameter was measured using an MKC-25 digital micrometer (LLC “Kalibron”, Chelyabinsk, Russia) by clamping the fiber between its measurement surfaces.

### 2.5. Filtration Studies

Core sample preparation and filtration studies were conducted in accordance with the requirements described in [59].

**Figure 6:**
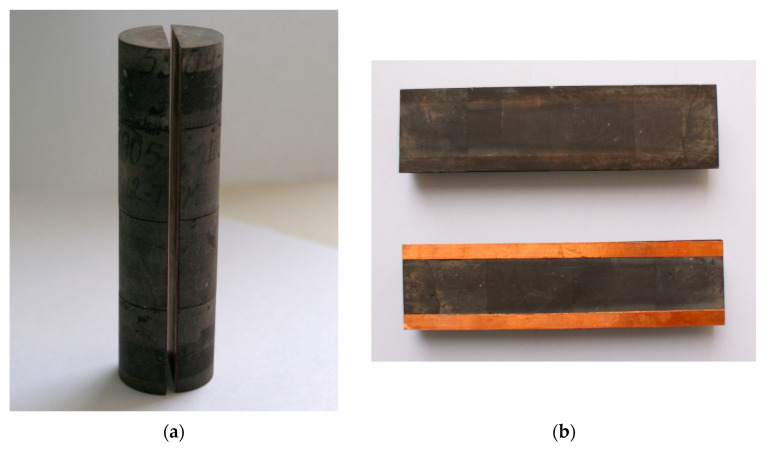

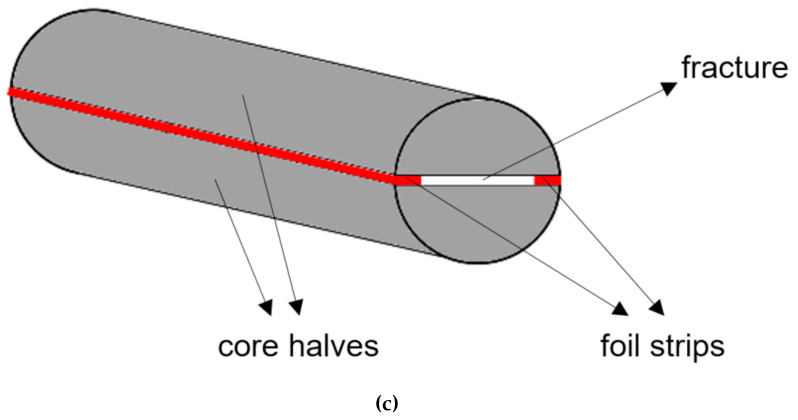
Photo of the ideal fracture model: (**a**) photo of the sawn core; (**b**) photo of the sawn halves of the core with glued foil strips; (**c**) scheme of an ideal fracture [49].

When creating the ideal fracture model (slot model) shown in Figure 6, natural core samples were used to adequately reproduce natural wettability conditions. The cylindrical core samples were then glued together to form a composite porous medium model with a length of at least 11.2 cm. These composite porous medium models were cut lengthwise, and the halves were matched to maintain the slot model’s cylindrical shape. After polishing the contacting surfaces of the slot model, foil strips of specified thickness were attached to one half to achieve the desired fracture aperture. The parameters of the fabricated ideal fracture model were as follows: length—11.2 cm; width—1.7 cm; nominal gap (slot aperture)—50, 100, and 650 μm; spatial orientation—horizontal.

Filtration tests were conducted using the SMP FES-2R unit (LLC «Kortech», Mytishchi, Russia). The unit specifications are as follows: core model length, 100–300 mm; core temperature range, 25–150 °C; maximum rock pressure, 70 MPa; and maximum formation pressure, 55 MPa.

**Figure 7:**
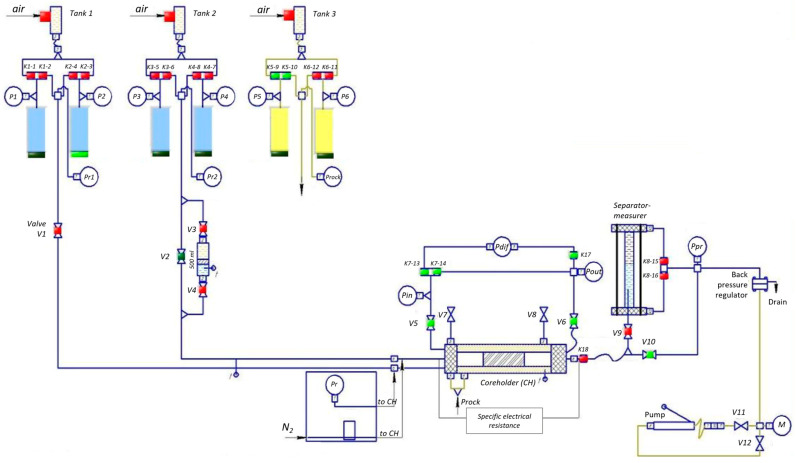
Schematic diagram of experimental unit SMP FES-2R [45].

The SMP FES-2R filtration unit (Figure 7) consists of the main unit (working table) and the electronics unit. The main unit contains the following:

- Core holder with an electric heater;
- The main hydraulic system, which performs the functions of formation fluid supply and determination of their volumes (the main system includes two sub-systems corresponding to two fluid phases);
- An auxiliary hydraulic system designed to create rock (crimp) pressure;
- A system for creating back pressure during filtration;
- The sensors of the control and measurement system;
- The distribution gas combs of the pneumatic valve control system connected to the air line at 5.5–6.5 atm.

To measure and regulate the temperature, the system “thermocouples (thermoresistance)–meter-temperature regulator–computer–power relay-regulator–heating elements” is used, further separated into a temperature regulation system.

In the electronics block, the following are installed: HART modem, the input–output unit of pressure sensors, the blocks (drivers) of the stepper motor control of high-pressure pump drives, power supply units, the input–output unit of pneumatic comb control, a personal computer system unit, a digital immittance meter, an electronic ultrasound unit and data acquisition board (oscilloscope), and a MOXA UPORT 1650-8 expansion board for eight COM ports.

Before each experiment, the surface of the slot model was carefully prepared, cleaned of contaminants, and washed with water and an alcohol solution. The ideal fracture model was placed in the core holder of the core filtration test setup, and the required volume of hydrogel was filtered.

The experiments were conducted at a temperature of 29 °C. For each model, the first stage involved water filtration in the forward direction with a volume of at least 10 cm^3^ until pressure gradient stabilization. Then, oil filtration was performed in the forward direction with a volume of at least 10 cm^3^ until pressure gradient stabilization. At the next stage, water was filtered in the forward direction at a constant flow rate (0.1, 0.5 cm^3^/min) with a volume of at least 10 cm^3^ until pressure gradient stabilization, with water permeability determined at each stage. Next, a plugging composition was injected into the ideal fracture model in the direction opposite to the initial one, in a volume not exceeding 10 cm^3^. After this, the system was allowed to settle under static conditions for at least 24 h. At the following stage, water was injected in the forward direction at a constant flow rate (0.1, 0.5 cm^3^/min) with a volume of at least 10 cm^3^ until pressure gradient stabilization, with water permeability and maximum pressure gradient determined at each stage. As a result, the residual resistance factor (RRF) was calculated—the ratio of water pressure drops after composition injection to the pressure drop before reagent exposure:

(1) $$RRF=\frac{dP_{i}}{dP_{1}}$$

where dP_i_—pressure drop after gel injection, MPa; dP_1_—pressure drop before gel injection, MPa.

It is important to note that rheological experiments and low-pressure filter press tests were conducted at ambient room temperature, while filtration experiments using the ideal fracture model were performed at 29 °C to match actual reservoir temperature conditions during field tests.

The first two types of studies (rheological and filter press tests) were carried out for the optimization of water shutoff compositions, whereas the filtration unit tests were designed to closely simulate real geological and reservoir conditions.

### 2.6. Field Test

**Figure 8:**
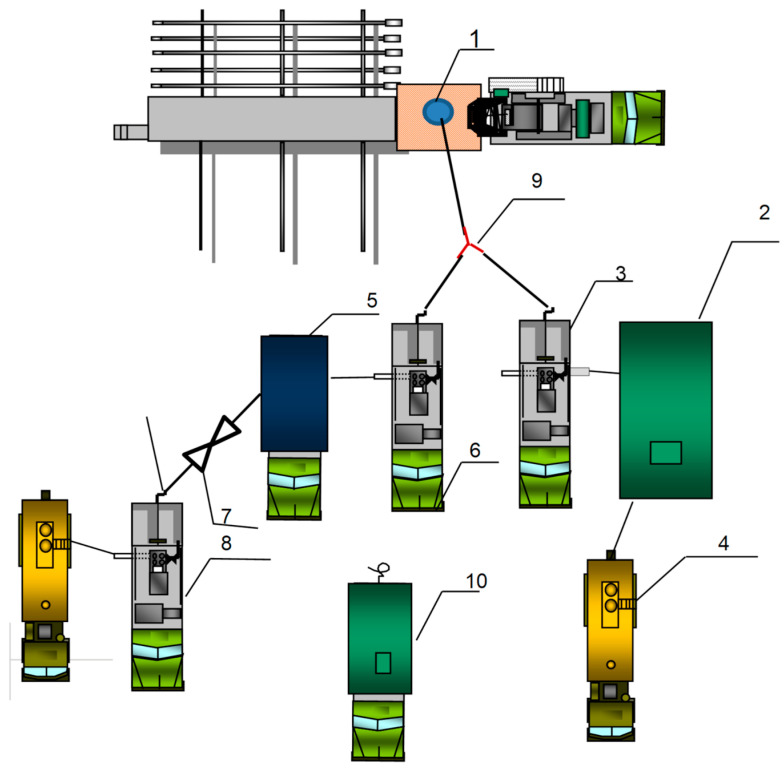
Special equipment layout for water control operation in a production well (1—wellhead; 2, 5—mixing–homogenizing units (MHUs); 4—fresh water tank trucks; 3, 6, 8—CA-320 cementing units; 7—ejector; 9—tee connection; 10—mobile steam-field unit (SFU).

Field trials for water shutoff operations were conducted at a well in the Republic of Tatarstan oilfield. The test objectives focused on reducing water cut in the fractured–porous reservoir of the Bashkirian horizon. To enhance the penetration depth of the plugging material into the near-wellbore zone, polymer composite injection was performed in two stages: initially with 30 m^3^ of foam–gel mixture (described in [45]), and the operation continued with the injection of 5.5 m^3^ of gel–fiber composite followed by water displacement. Figure 8 shows the layout and piping diagram of specialized equipment used for the water control operation in the production well. Preparation of 15 m^3^ quantities for each component of the foam–gel composition was performed in mixing–homogenizing units (MHUs) (units 2 and 5). Injection was carried out at constant rate using two CA-320 cementing units (units 3 and 6) through a tee connection (9). The CA-320 unit (8) then metered components of the fiber composite into the MHUs (5) via an ejector (7). After achieving homogeneous composition in the MHU (5), injection into the formation was performed using the CA-320 unit (6). The treatment was completed with 4.8 m^3^ of water displacement and a 24 h shut-in period. Notably, during winter operations with fresh water, having a mobile steam-field unit (SFU) (10) on location was mandatory to prevent freezing of surface piping in case of unplanned injection stoppage.

## 3. Results and Discussion

### 3.1. Rheological Studies

The first series of experiments investigated the rheological properties of fiber-reinforced gel composites (Table 1). The base matrix (BM) composed of polyacrylamide and crosslinking agents served as the reference system.

As evident from Table 1, the addition of PPF in concentrations ranging from 0.05% to 0.25% gradually enhances all rheological parameters, with the storage modulus increasing from 23.07 Pa for the BM to 28.63 Pa. A further increase in PPF concentration does not lead to significant changes in rheological properties. A similar trend is observed for PANF: the storage modulus increases from 0.10% to 0.20% concentration, while at higher concentrations (0.25–0.30%) it shows a slight decrease. These results suggest that the optimal fiber content for both PPF and PANF in the composite falls within the 0.15–0.20% range. When chrysotile microfibers were incorporated at concentrations of 0.5–2.0%, the storage modulus demonstrated a consistent increase from 29.38 Pa to 33.65 Pa.

**Figure 9:**
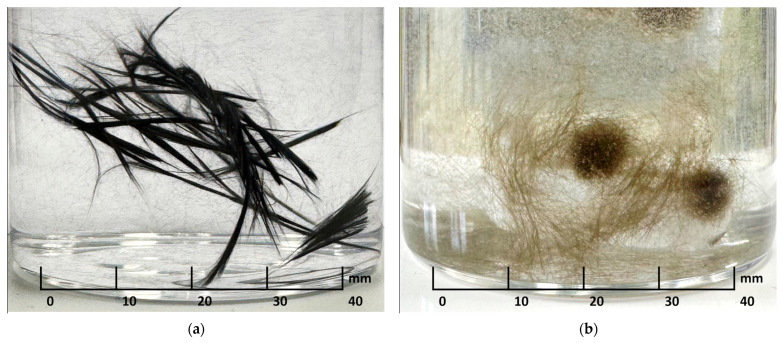
Visual manifestation of hydrophobicity: (**a**) carbon fibers; (**b**) basalt fibers.

It should be noted that homogeneous composite materials cannot be obtained with carbon fibers and basalt fibers due to a strong hydrophobic effect, where the fibers are repelled by the aqueous medium and aggregate into long bundles (CFs) or spherical clusters (BFs) at the bottom of the container. BFs acquire hydrophobic properties during the fiber production process: to prevent them from sticking together, their surface is treated with sizing agents (“oiled”), which promotes hydrophobic behavior in aqueous environments (Figure 9a,b).

**Figure 10:**
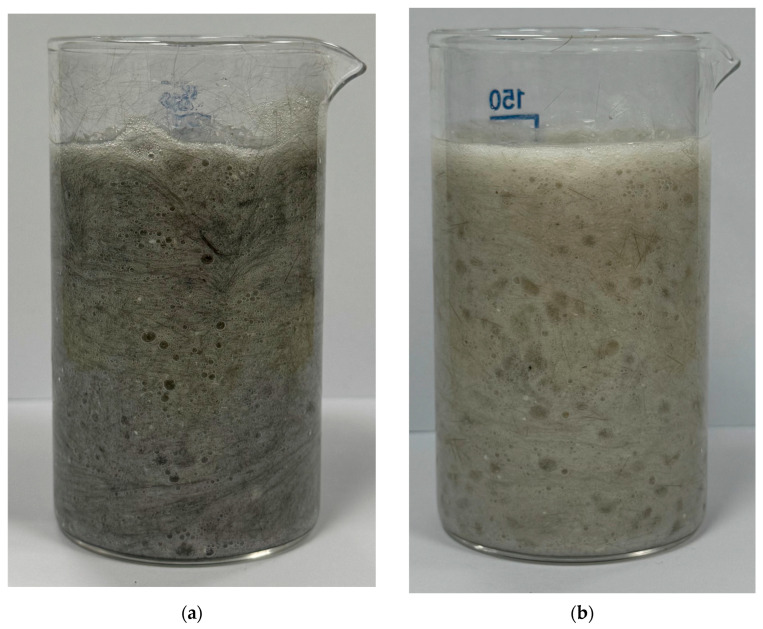
Suppression of hydrophobic effect using chrysotile suspension: (**a**) carbon fibers and chrysotile in BM; (**b**) basalt fibers and chrysotile in BM.

The hydrophobic effect in CF- and BF-based composites can be avoided by creating binary composite materials through preliminary dispersion of 1.5% chrysotile in gelant. Chrysotile (magnesium hydrosilicate) forms multiple hydrogen bonds with the aqueous solution. Subsequent addition of CF or BF results in their uniform dispersion within the chrysotile–gelant suspension (Figure 10a,b).

The rheological properties of binary and ternary fiber-reinforced composites in the base matrix are presented in Table 2.

From Table 2, it is evident that the composite containing 1.5% chrysotile and 0.2% CF shows a significantly higher storage modulus (34.74 Pa) compared to single-fiber compositions. The composite with BF at similar loading demonstrates somewhat inferior performance, specifically exhibiting storage modulus of 29.39 Pa. For oxidized CF and CF with silica surface modification (~20%) via sol–gel deposition, the storage modulus values are even lower: 27.55 Pa and 27.31 Pa, respectively.

The observed reduction in the linear viscoelastic region upon fiber incorporation into the hydrogel matrix will have negligible impact on water shutoff efficiency, since we inject a gellant (i.e., non-matured gel). After gelation occurs in situ, the primary parameter characterizing viscoelasticity becomes the elastic modulus (G′).

Our field experience in both vertical and horizontal wells—including operations under elevated drawdown pressures [45]—demonstrates no gel mass washout. This indicates that within a certain LVR, these compositions maintain stability under shear stresses.

For CF with electrochemical surface modification by silicon dioxide (60%), the storage modulus reaches 34.62 Pa—nearly identical to pristine CF. The combinations of CF (0.1%) with PPF (0.1%) and CF (0.1%) with PANF (0.1%) yielded composites with the highest rheological properties. Specifically, the PPF-containing composition showed a storage modulus of 35.68 Pa, while the PANF-containing one reached 46.50 Pa. Significant increases were also observed in the loss modulus (up to 22.01 and 26.36 Pa, respectively) and complex modulus (up to 41.93 and 53.46 Pa). The synergistic effect in ternary systems containing millimeter-length fibers (CF, PPF, and PANF) is attributed to van der Waals interactions with the polymer backbone, while chrysotile reinforces the composite through hydrogen bonding with functional groups. It should be noted that the experiments did not confirm our hypothesis about improved rheological properties through CF sizing. Composites with oxidized CF or SiO_2_-coated fibers demonstrated inferior properties compared to untreated CF.

It should be noted that the SiO_2_ coating methods differ from each other and form distinct structures on the fibers, as evident in Figure 2 and Figure 3. As mentioned earlier, acid treatment etches the carbon fiber surface, increasing roughness. A roughened surface enhances contact area and adhesion with the polyacrylamide hydrogel matrix.

The likely negative impact of SiO_2_ coatings on the rheological properties of the composites can be attributed to untreated carbon fibers maximizing interaction with the polymer backbone through van der Waals forces. The fiber surface treatments aimed to promote hydrogen bonding with the polymer’s functional groups. However, since these interactions occur in an aqueous environment, primary hydrogen bonds form preferentially with water molecules. This reduces the hydrophobic effect (entropically driven) and, consequently, weakens van der Waals interactions. It is well established that van der Waals forces between carbon fibers and hydrocarbon chains of carbon-backbone polymers arise from short-range dispersion forces.

Coating thickness also influenced composite properties, though this aspect requires further study. Notably, modified carbon fibers could reinforce other hydrogel matrices (e.g., polysaccharide or polyacrylamide/polysaccharide systems) by forming hydrogen bonds, thereby enhancing composite structure.

The next series of rheological experiments was conducted using fiber-dispersed composites with a filler content of 1.0%. Mechanically activated shungite mineral and zinc oxide were selected as the dispersed components. The unique composition of shungite [60,61] suggested that the silica (57%) contained within it could strengthen the composite structure after mechanical activation by forming hydrogen bonds with the polymer’s functional groups [62], while the carbon components (30% of shungite) would contribute through van der Waals interactions with the polymer’s main chain. Zinc oxide was chosen because it is used in fibrous composites for water shutoff [32]. The rheological properties of the gel–fiber-dispersed composites are presented in Table 3.

As evident from Table 3, the addition of 1% mechanically activated shungite to composites containing 0.15% PANF or PPF results in only a marginal increase in storage modulus—observed exclusively for PANF-based systems (up to 31.00 Pa). An identical trend was noted with zinc oxide dispersions, where no significant reinforcement effect was achieved.

In fiber-dispersed composites containing chrysotile (1.5%) and CF (0.2%), the addition of shungite and zinc oxide increased the storage modulus to 35.31 Pa and 42.76 Pa, respectively. Thus, it can be concluded that shungite and zinc oxide additives only partially met expectations: their rheological properties were comparable to ternary fiber composites like CF–chrysotile–PPF.

Although the increase in elastic modulus is modest in compositions containing mechano-activated shungite, this enhancement will promote tighter particle packing within fractures and improve water breakthrough resistance during flowback. This approach aligns with conventional well killing operations using particulate suspensions in xanthan solutions, where optimal particle sizing follows Vickers’ ideal packing theory [63]. We have successfully implemented this methodology in fractured reservoir well control scenarios.

**Figure 11:**
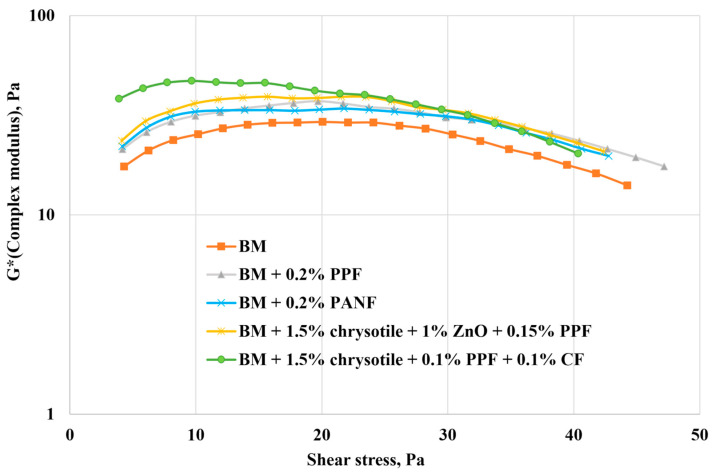
Dependencies of the complex modulus on shear stress.

Figure 11 presents the complex modulus (G*) versus shear stress dependencies for the most effective compositions selected for filtration studies using the idealized fracture model.

### 3.2. Filtration Studies

Filtration tests were performed using an FLR-2 low-pressure filter press and an SMP FES-2R filtration unit. The filter cell volume was 156 mL, containing 150 mL of the test composite. The gel maturation time was 48 h. As a reference point, we measured the filtration time of the base gel through a slot with the following parameters: length 30 mm, width 300 μm, corresponding to large-aperture fracture dimensions. Under experimental conditions, the BM filtered through in 15 s, demonstrating the unmodified gel’s inability to block fractures (Table 4).

**Figure 12:**
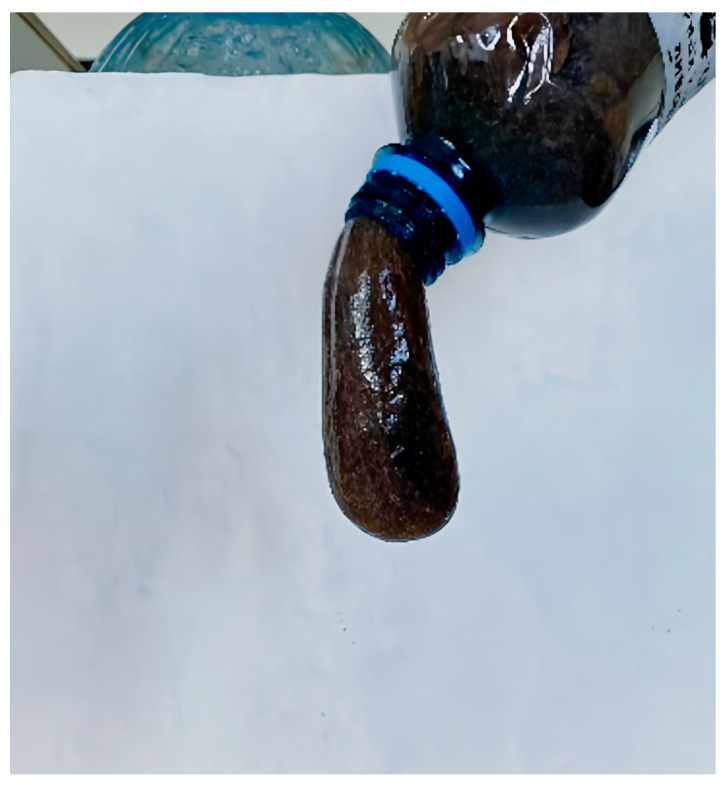
Appearance of the composite (composition №7, Table 4).

The addition of 0.05% PPF led to the filtration of 80 mL of composite through the slot in 30 min. Increasing the content of these fibers to 0.15% reduced the passage of composite through the slot (30 mL of composite in 30 min). Raising the PPF content to 0.20% and 0.25% decreased the filtrate volume to 5 mL of composite in 30 min. The use of composites with three fibers, such as chrysotile (1.5%), PPF/PANF (0.1%), and CF (0.1%), resulted in an even smaller volume of composite passing through the slot in 30 min: 4 mL of PPF and 2.5 mL of PANF. It should be noted that the results of the rheological study and filtration testing on the low-pressure filter press generally coincide. A photograph of one of the best composites (composition 7, Table 4) is shown in Figure 12.

The results of filtration studies conducted using the idealized fracture model are presented in Table 5.

Filtration in an ideal fracture model was performed by injecting 0.3 pore volumes (PV) of a composite containing 0.15% PPF into a fracture with an aperture of 50 μm. During reverse water filtration at flow rates of 0.1 cm^3^/min and 0.5 cm^3^/min, the RRF measured 24.72 units and 7.23 units, respectively.

When the fracture aperture was increased to 100 μm, injection of 0.3 PV of a composite containing two fiber types—chrysotile (1.5%) and PPF (0.15%)—resulted in RRF values of 167.3 units (0.1 cm^3^/min) and 162.4 units (0.5 cm^3^/min).

For a 650 μm fracture aperture, a composite incorporating three fiber types—carbon fiber (0.1%), PAN fiber (0.1%), and chrysotile (1.5%)—demonstrated RRF values of 82.3 units (0.1 cm^3^/min) and 9.76 units (0.5 cm^3^/min) during reverse water filtration.

The implementation of the best-performing composites identified through rheological studies (Figure 11)—representing the three investigated groups (monofiber-reinforced, hybrid fiber with 2–3 fiber types, and gel–fiber-dispersed composites)—yielded high residual resistance factors in filtration tests (Table 5), even when the fracture aperture increased by an order of magnitude.

Based on the results of our comprehensive rheological and filtration laboratory studies, the developed composites can be recommended for application as follows (Table 6).

The selection of ideal fracture aperture parameters was based on median values characteristic of the fracture network in the target reservoir. When injecting uncrosslinked polymer solution (gellant) into fractured–porous formations, the fluid penetrates natural fracture systems with variable apertures and tortuosity. The gellant’s rheological properties—resembling a high-viscosity Newtonian fluid—ensure uniform distribution even in complex geometries. This suggests complete fracture volume filling and subsequent formation of a continuous gel barrier under actual reservoir conditions with variable apertures. Consequently, our idealized fracture model accurately represents the key isolation mechanism in terms of gel continuity.

Regarding barrier stability, fracture wall roughness enhances gel adhesion according to polymer–rock mechanical interaction data. The increased contact area between gel and rough fracture walls elevates the composite’s hydrodynamic resistance to shear stresses and prevents gel washout during production.

Thus, despite geometric simplifications, our model correctly accounts for two critical physical processes: complete fracture volume saturation by gellant and adhesion enhancement on rough surfaces. These factors confirm that fracture geometry idealization does not overestimate isolation efficiency under field conditions.

### 3.3. Field Test

**Figure 13:**
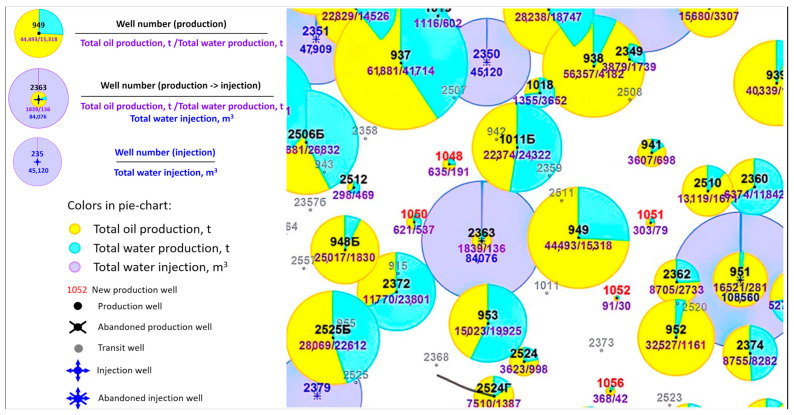
Total production map: Zyuzeyevskoye field, well 949.

Field water shutoff operations were carried out at production well 949 of the Zyuzeyevskoye field (Bashkirian horizon). A sharp increase in water cut was observed at this well—from 40% (2017) to 96% (July 2023). It was revealed that shutting down injection at the nearest injection well 2363 rapidly reduces water cut in production well 949, which indirectly indicates injected water breakthrough. Indeed, as can be seen from the total production map (Figure 13), well 949 is located within the zone of influence of injection well 2363.

**Figure 14:**
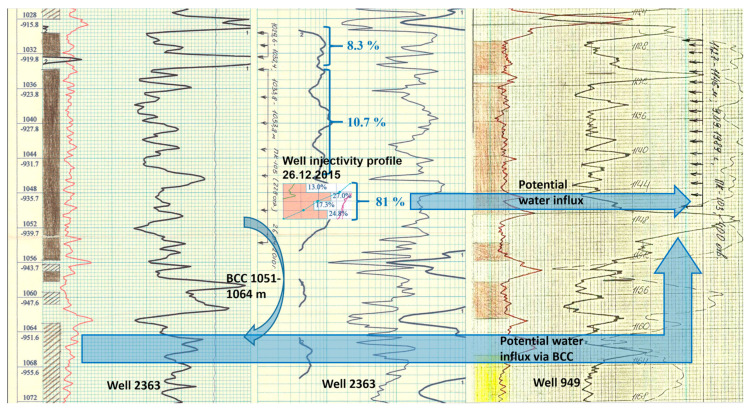
Comparison of radioactive logging data and injectivity profile data from injection well 2363.

Since no production logging (PL) was conducted in well 949 to identify water breakthrough sources, we compared gamma-ray logs from wells 2363 and 949 along with the injectivity profile of well 2363 (Figure 14) during the workover preparation analysis. Based on this comparison, it was hypothesized that excess injected water could enter both through the base of the perforated formation interval and via behind-casing chanelling (BCC) from injection well 2363 (blue arrows in Figure 14 indicate the water influx pathways).

**Figure 15:**
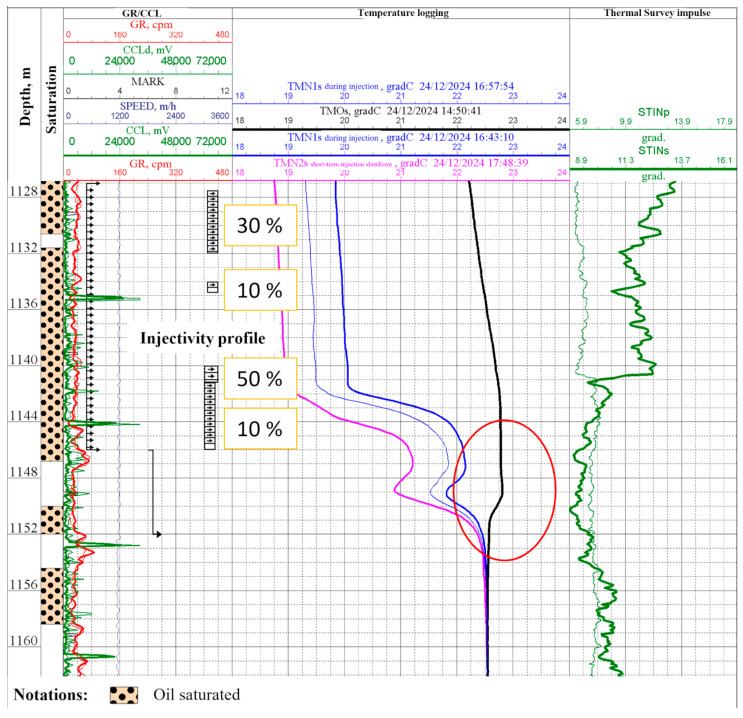
PL log sheet dated 24 December 2024: well 949.

PL was conducted immediately prior to the workover operation on 24 December 2024 to evaluate the injectivity profile. The results revealed behind-casing channeling in the 1146–1155 m interval, as evidenced by temperature logging measurements in the shut-in well 949 (Figure 15, marked with red oval), thereby confirming our initial hypotheses.

The water shutoff operation was performed on 26 December 2024.

**Figure 16:**
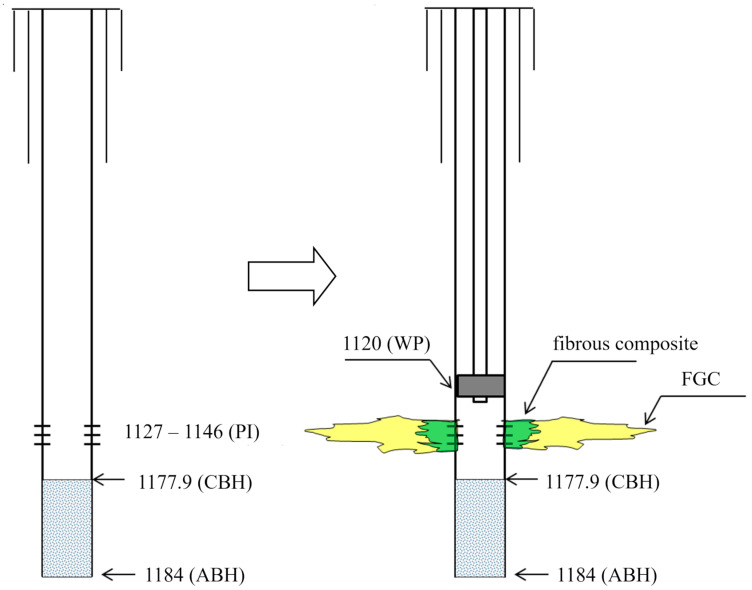
Process of conducting workover and isolation operations (WIOs) for water shutoff at well 949. The process of performing workover and isolation operations for water shutoff at well 949. (WP—workover packer; PI—perforation interval; CBH—current bottomhole; ABH—artificial bottomhole; FGC—foam–gel composition).

For water isolation in the fractured–porous carbonate formation of the Bashkirian horizon, it was decided to first inject a foam–gel composition that easily filters before gel maturation, followed by a second stage of fibrous composite injection. The well treatment schematic is shown in Figure 16.

Table 7 presents the operational parameters of well 949 before and after treatment. It should be noted that prior to implementing the water inflow control technology, the injectivity was 576 m^3^/day at 0 atm on the mobile pumping unit pressure gauge.

The isolation work was performed through the existing perforation interval. A total of 30 m^3^ of the foam–gel composition was injected into the formation at 0 atm, followed by 5.5 m^3^ of fibrous composite containing 1.7% polyacrylamide and 0.15% PPF. The pressure increased to 35 atm by the end of injection and reached 100 atm after displacement with technical water.

As shown in Table 7, the water cut decreased to 35% before the startup of injection well 2363, but it increased to 50% after the reservoir pressure maintenance system was activated. Based on these results, the subsoil user was recommended to implement conformance control measures in well 2363 to stabilize the injectivity profile, which would reduce the produced water cut to 35–40%.

### 3.4. Environmental Impact, Sustainability, and Risk Assessment of Additives

Chrysotile asbestos is classified as hazardous to human health, with confirmed risks of cancer (e.g., mesothelioma, lung cancer) and other diseases due to its fibrous structure [64]. However, its use in non-friable products such as polymer composites may reduce these risks when strict safety measures are implemented: for example, preventive measures including wet processing methods to suppress dust generation, the use of personal protective equipment (PPE), and adherence to hygiene protocols are critical during field operations [65,66]. In our study, the chrysotile–polymer system was designed to encapsulate fibers within a crosslinked matrix, minimizing their release potential. This approach aligns with recommendations from the International Chrysotile Association, which emphasizes the importance of controlled lifecycle management for safe handling [66].

Scientific data indicate that ZnO, whether in nanoparticle or microparticle form, exhibits a high safety profile suitable for petrochemical applications. Toxicological studies have not identified significant cytotoxicity, genotoxicity, or carcinogenicity associated with ZnO, nor have unexpected toxic effects been observed following subcutaneous or intravenous administration. A key safety advantage of ZnO is its low solubility, which prevents dermal penetration and minimizes systemic absorption risks during industrial handling. While inhalation exposure should still be controlled through standard dust suppression methods, overall toxicity remains low. In the petroleum industry, ZnO serves as a stable, non-reactive additive in lubricants, corrosion inhibitors, and catalytic processes, forming no hazardous degradation products due to its thermal and chemical stability [67].

Safety studies demonstrate that shungite is generally non-toxic, with no significant cytotoxicity reported in cell line studies. Its use in food and cosmetic industries further supports its low-risk profile when properly processed. However, as a natural mineral, shungite composition may vary, with some deposits containing trace heavy metals, necessitating quality control to minimize contamination risks. In industrial settings, the primary safety concern is dust inhalation, which may pose respiratory hazards similar to other fine mineral powders. Risk mitigation requires proper handling, including dust suppression measures and PPE [68].

Regarding disposal (chrysotile, ZnO, mechanoactivated shungite), the technology is waste-free, as all components are injected into the formation as composites and are not returned to the surface.

## 4. Conclusions

Oscillatory test results showed that adding fibrous and dispersed particles significantly increases the elastic modulus compared to the base composition (G′ = 23 Pa). The following formulations were selected for further studies:

- Single-fiber systems: composition with 0.15–0.2% PPF/PANF, showing G′ ≈ 28 Pa;
- Multi-fiber systems: best result (G′ = 46.5 Pa) with 1.5% chrysotile + 0.1% PANF + 0.1% CF;
- Fiber-reinforced gel-dispersed composites: optimal results (G′ ≈ 30 Pa) with 0.2% PPF/PANF + 1.5% chrysotile + 1% dispersed filler (shungite/zinc oxide).

Filtration tests showed the following:

- At a 0.1 cm^3^/min flow rate: RRF values of 24.72, 167.3, and 82.3 for fracture widths of 50, 100, and 650 μm, respectively;
- At a 0.5 cm^3^/min flow rate: RRF values of 7.23, 162.4, and 9.76 for the same fracture widths.

Thus, oscillatory rheometry and slot-flow filtration methods allowed us to develop a series of water-isolating compositions for fractured carbonate reservoirs based on polymer–fiber composites. These compositions cover practically the entire range of fracture widths found in oil reservoirs. Field tests at a well in the Volga-Urals region confirmed the approach’s validity. Standard equipment was used for pumping the fiber compositions without complications, despite winter conditions. Further research will focus on polysaccharide hydrogel matrices that eliminate hazardous components for human health and the environment.

In conclusion, it should be noted that fibrous composites incorporated within a polymer–gel matrix serve as an effective tool for water production control in fractured reservoirs. By varying the composition of fibers and particulate fillers embedded in the gel matrix, it becomes possible to regulate excessive water influx in naturally fractured reservoirs. Fiber reinforcement enhances the composite’s mechanical strength, ensures effective water flow blockage, and prevents washout of the sealing material during well completion operations.

## Figures and Tables

**Table 1:** Rheological parameters of BM composites with monofilaments.

No.	Composition	G′, Pa	G″, Pa	G*, Pa	Crossover, Pa	LVR, Pa
1	BM	23.07	11.72	25.88	45.27	31.36
2	BM + 0.05% PPF	24.23	12.28	27.17	43.99	30.30
3	BM + 0.10% PPF	26.62	14.19	30.16	41.34	26.99
4	BM + 0.15% PPF	27.34	14.81	31.10	41.03	25.97
5	BM + 0.20% PPF	27.52	13.88	30.82	46.45	32.95
6	BM + 0.25% PPF	28.63	15.23	32.44	46.16	32.82
7	BM + 0.30% PPF	28.58	15.35	32.45	45.72	31.14
8	BM + 0.10% PANF	23.85	12.54	26.95	44.82	32.33
9	BM + 0.15% PANF	26.22	13.10	29.32	45.73	31.60
10	BM + 0.20% PANF	27.77	13.26	30.78	45.04	30.63
11	BM + 0.25% PANF	26.39	12.84	29.35	45.91	32.05
12	BM + 0.30% PANF	26.27	12.97	29.30	43.03	29.39
13	BM + 0.5% chrysotile	29.38	16.31	33.61	42.72	28.11
14	BM + 1% chrysotile	31.08	18.19	36.02	40.22	26.97
15	BM + 2% chrysotile	33.65	19.38	38.84	41.14	27.50

**Table 2:** Rheological parameters of BM composites with two and three fiber types.

Composition	G′, Pa	G″, Pa	G*, Pa	Crossover, Pa	LVR, Pa
BM	23.07	11.72	25.88	45.27	31.36
BM + 1.5% chrysotile + 0.2% BF	29.39	15.11	33.05	42.12	30.43
BM + 1.5% chrysotile + 0.2% CF	34.74	18.43	39.33	39.94	26.43
BM + 1.5% chrysotile + 0.2% CF oxidized	27.55	15.93	31.84	28.75	19.53
BM + 1.5% chrysotile + 0.2% CF/SiO_2_ (sol–gel deposition)	27.31	18.22	32.99	25.43	14.90
BM + 1.5% chrysotile + 0.2% CF oxidized/SiO_2_ (electrochemical deposition)	34.62	18.57	39.29	39.23	26.42
BM + 1.5% chrysotile + 0.1% BF + 0.1% CF	25.67	13.63	29.07	30.96	21.38
BM + 1.5% chrysotile + 0.1% PPF + 0.1% CF	35.68	22.01	41.93	37.15	22.01
BM + 1.5% chrysotile + 0.1% PANF + 0.1% CF	46.50	26.36	53.46	44.39	26.68

**Table 3:** Rheological testing results of gel–fiber-dispersed composites.

Composition	G′, Pa	G″, Pa	G*, Pa	Crossover, Pa	LVR, Pa
BM	23.07	11.72	25.88	45.27	31.36
BM + 1.5% chrysotile + 1% shungite + 0.2% CF	35.31	18.94	40.07	40.75	28.05
BM + 1.5% chrysotile + 1% ZnO + 0.2% CF	42.76	22.63	48.37	43.36	30.17
BM + 1% shungite + 0.15% PANF	31.00	16.82	35.27	46.28	31.63
BM + 1% shungite + 0.15% PPF	25.21	13.93	28.81	41.06	29.47
BM + 1.5% chrysotile + 1% shungite + 0.15% PPF	30.96	16.98	35.31	42.95	30.43
BM + 1% ZnO + 0.15% PANF	28.37	14.24	31.75	49.48	33.25
BM + 1% ZnO + 0.15% PPF	26.82	13.90	30.21	44.73	31.19
BM + 1.5% chrysotile + 1% ZnO + 0.15% PANF	31.15	15.86	34.96	45.25	31.95
BM + 1.5% chrysotile + 1% ZnO + 0.15% PPF	28.89	14.96	32.53	39.66	28.78

**Table 4:** Low-pressure filter press test results for composites.

No.	Composition	Filtration Time, min	Filtrate Volume, mL
1	BM	0.25	150
2	BM + 0.05% PPF	30	80
3	BM + 0.15% PPF	30	30
4	BM + 0.20% PPF	30	5
5	BM + 0.25% PPF	30	5
6	BM + 0.10% PPF + 1.50% chrysotile + 0.10% CF	30	4
7	BM + 0.10% PANF + 1.50% chrysotile + 0.10% CF	30	2.5

**Table 5:** Filtration studies results.

Fracture Aperture, μm	Composition	Flow Rate, m^3^/min	RRF
50	BM + 0.15% PPF	0.1	24.72
		0.5	7.23
100	BM + 0.15% PPF + 1.50% chrysotile	0.1	167.3
		0.5	162.4
650	BM + 0.10% PANF + 1.50% chrysotile + 0.10% CF	0.1	82.3
		0.5	9.76

**Table 6:** Application of the developed composites.

Fracture Aperture, μm		Composition
20–100		0.15–0.2% monofilament fibers (PPF/PANF)
100–300	(a)	(a) 0.2% PPF/PANF + 1.5% chrysotile
	(b)	(b) 0.2% PPF/PANF + 1.5% chrysotile + 1% particulate filler (shungite/zinc oxide)
> 300		0.1% CF + 0.1% PPF/PANF + 1.5% chrysotile

**Table 7:** Well 949 operational parameters before and after workover isolation operations.

Well Operation Mode	Liquid Flow Rate, m^3^/day	Oil Flow Rate, m^3^/day	Water Cut,%	Bottomhole Pressure, atm
Before WIO	17	0.2	99	8
After WIO (as of 1 January 2025) (reservoir pressure maintenance system not activated)	8	4.8	35	5
After WIO (as of 20 January 2025) (reservoir pressure maintenance system activated)	7.5	3.5	50	6
After WIO (as of 1 March 2025)	7.4	2.5	63	4

## Data Availability

The original contributions presented in this study are included in the article. Further inquiries can be directed to the corresponding authors.

## Abbreviations

The following abbreviations are used in this manuscript:

PANF	polyacrylonitrile fiber
PPF	polypropylene fiber
BF	basalt fiber
CF	carbon fiber
PAM	polyacrylamide
MFMC	mass fraction of the main component
BM	base matrix
SEM	scanning electron microscope
LVR	linear viscoelastic region
RRF	residual resistance factor
BCC	behind-casing channeling
PL	production logging
WIO	workover and isolation operation
PPE	personal protective equipment
